# Integrating Geophysical and Multispectral Data to Delineate Homogeneous Management Zones within a Vineyard in Northern Italy

**DOI:** 10.3390/s19183974

**Published:** 2019-09-14

**Authors:** Bianca Ortuani, Giovanna Sona, Giulia Ronchetti, Alice Mayer, Arianna Facchi

**Affiliations:** 1 Department of Agricultural and Environmental Sciences–Production, Landscape, Agroenergy, Università degli Studi di Milano, via Celoria 2, 20133 Milan, Italy; alice.mayer@unimi.it (A.M.); arianna.facchi@unimi.it (A.F.); 2 Department of Civil and Environmental Engineering, Politecnico di Milano, P.zza Leonardo da Vinci 32, 20133 Milan, Italy; giovanna.sona@polimi.it (G.S.); giulia.ronchetti@polimi.it (G.R.)

**Keywords:** geophysical data, multispectral data, thermal imagery, data fusion, homogeneous management zones, crop water stress

## Abstract

Soil electrical conductivity (EC) maps obtained through proximal soil sensing (i.e., geophysical data) are usually considered to delineate homogeneous site-specific management zones (SSMZ), used in Precision Agriculture to improve crop production. The recent literature recommends the integration of geophysical soil monitoring data with crop information acquired through multispectral (VIS-NIR) imagery. In non-flat areas, where topography can influence the soil water conditions and consequently the crop water status and the crop yield, considering topography data together with soil and crop data may improve the SSMZ delineation. The objective of this study was the fusion of EC and VIS-NIR data to delineate SSMZs in a rain-fed vineyard located in Northern Italy (Franciacorta), and the assessment of the obtained SSMZ map through the comparison with data acquired by a thermal infrared (TIR) survey carried out during a hot and dry period of the 2017 agricultural season. Data integration is performed by applying multivariate statistical methods (i.e., Principal Component Analysis). The results show that the combined use of soil, topography and crop information improves the SSMZ delineation. Indeed, the correspondence between the SSMZ map and the CWSI map derived from TIR imagery was enhanced by including the NDVI information.

## 1. Introduction

In agriculture an effective and efficient management of inputs (i.e., water and nutrients) is fundamental to make the crop production sustainable, for both the environment and economics. Knowledge about the plant-soil system is fundamental to achieve this goal. Since the nineties, the Precision Agriculture (PA) approach has required a detailed description of the variability at field scale of soil and plant properties, in order to apply water and nutrients with variable rates, according to the actual irrigation and nutrient requirements, which can be extremely different within a field because of the spatial variability of soil and plant properties [1]. Following this approach, not only the water and nutrient use efficiencies, but also the quantity and the quality of crop yield may improve. Site-specific management of water and nutrients in PA requires the delineation in the field of sub-regions with similar soil and crop characteristics affecting crop yield (Site Specific Management Zone, SSMZ) [2]. Intensive and relatively time-saving measurements of soil electrical conductivity (EC) through geophysical proximal soil sensors are among the most frequently used approaches in PA to delineate SSMZs [3,4,5,6,7]. Statistical procedures [8] are used to classify the EC maps derived by interpolation of the geophysical data, resulting in few sub-field zones to be managed separately. EC maps are considered to delineate SSMZs because EC is influenced by a combination of soil physical-chemical properties affecting crop yield, including soluble salts, clay content and mineralogy, soil water content, bulk density, organic matter, and pH. Finally, a strong relation has been shown to exist between EC and the total available water-holding capacity (AWHC) of soils [9,10,11,12,13,14]. The information on spatial variability of AWHC at field scale obtained from EC measurements may be extremely important to optimize irrigation [9,10,11], allowing the construction of an irrigation prescription map to be used to prescribe variable water amounts (i.e., variable rate irrigation, VRI) according to soil variability.

Ancillary data acquired by spectral sensors in the visible (VIS), near-infrared (NIR) and thermal infrared (TIR) regions shall be combined with EC data to characterize the soil spatial variability [15] and to improve the delineation of SSMZs [16,17]. Indeed, NIR reflectance from soil has been correlated with many soil properties, including total C, total N, water content and texture [18,19]. VIS-NIR and TIR images of the bare soil, acquired by sensors mounted on Unmanned Aerial Vehicles (UAVs), have been used to evaluate the spatial distribution of soil water content [20,21]. The Normalized Difference Vegetation Index (NDVI), calculated as a combination of VIS and NIR data, has been related with soil organic carbon [22,23]. An approach to improve the delineation of SSMZ by considering multi-sensor data describing both crop and soil variability is illustrated in [24]. Multi-sensor data included data acquired through geophysical sensors to describe soil variability and spectral reflectance data derived from satellite imagery to describe crop variability through the construction of vegetation indices maps. The most recent literature stresses the importance to consider multi-sensor data to optimally delineate SSMZs [25,26,27,28,29].

In non-flat areas, as often is the case with vineyards, topography can influence soil water conditions and consequently crop yield and field zonation; in this situation SSMZ delineation may be improved by integrating geophysical soil monitoring data with topography data [12]. The authors of [12] additionally emphasized how spectral vegetation indices (mostly NDVI), constructed from reflectance data acquired through proximal sensing, UAV, aircraft or satellite imagery, are useful in zoning vineyards when the crop behavior is different from year to year because of important interactions between soil and climate influencing the vine vigor.

The main objective of this work is the implementation of data fusion procedures to delineate a SSMZ map in a vineyard of 1.5 ha located in Franciacorta (BS), since this information is crucial for the elaboration of irrigation prescription maps to be used for the design and/or the management of variable-rate irrigation systems. In particular, this study shows how the different types of data which can be involved in the SSMZ delineation are acquired and analyzed, and finally integrated through a data-fusion approach. To assess the reliability of the SSMZ maps obtained from different types of data, each of them was compared with data acquired by a thermal infrared (TIR) survey carried out during a hot and dry period of the 2017 agricultural season, in a phase of the crop phenology during which the vine is normally most sensitive to water stress. Data collected during the TIR survey were used to produce a crop water stress index (CWSI) map, which was demonstrated to be well correlated with the final crop yield [30,31,32,33].

## 2. Materials and Methods

### 2.1. Study Area

**Figure 1:**
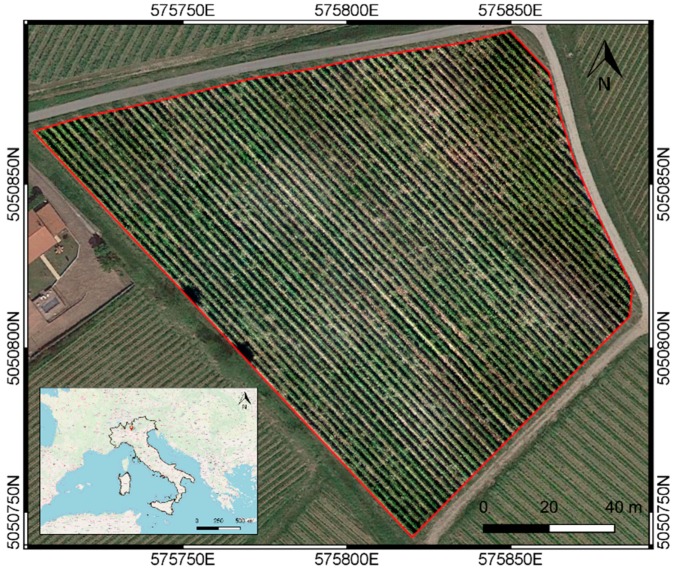
The experimental site; Coordinate Reference System (CRS): WGS84/UTM zone 32 N. Map data: ©OpenStreetMap contributors.

The experimental site is a rain-fed vineyard of 15,000 m^2^ located in Franciacorta (Erbusco, 575,813 E, 5,050,828 N, Northern Italy), a rolling hills area south-east of Lake Iseo (Figure 1). Soils at this site are sandy-loam in texture, according to the regional 1:250.000 soil map (http://www.geoportale.regione.lombardia.it). They belong to the land system of ‘intermediate moraine deposits’. In this area, the typic Paleudalf coarse loamy and poorly gravelly soils (CZO1), are associated with more skeletal soils, very deep, with moderately faster permeability and drainage (VBO1) [34].

**Figure 2:**
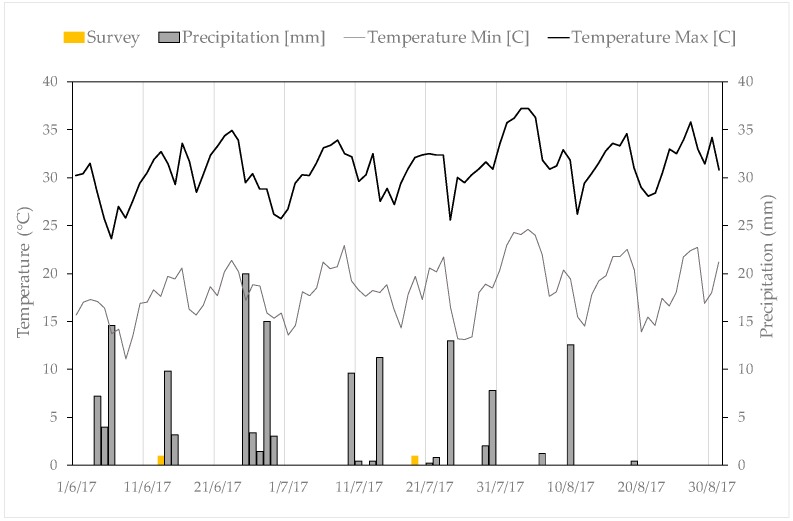
Precipitation and temperature daily data collected at the agrometeorological station of Erbusco, during the experimental period from June to August 2017.

In the Franciacorta region, the climate is continental, with the lake providing a mitigating effect in both summer and winter. Considering the average monthly values of the main agrometeorological variables registered at the Erbusco station (part of the Lombardy regional monitoring network), located 2 km far from the experimental site, for the period 2008–2018, it can be observed that the minimum and maximum monthly rainfall occur respectively in July (70 mm) and October (85 mm), while the minimum and maximum daily air temperatures vary, respectively, from 7 °C in October to 20 °C in July, and from 16 °C in October to 32 °C in July. Figure 2 shows the behavior of the main agrometeorological variables recorded at the Erbusco station during the experimental period June-August 2017.

### 2.2. Experimental Surveys

Different types of data were collected in the vineyard to describe all the factors affecting crop yield, related to the hydrological condition of the soil, as well as to the crop vigor and water status. The soil properties were detected through an electro-magnetic induction (EMI) sensor, while the topography of the vineyard and the crop properties were investigated through multispectral and thermal sensors mounted on UAV. Various combinations of these data (i.e., data fusion) were analyzed, to assess their effectiveness in improving the delineation of the SSMZs aimed at optimizing the crop yield (see Section 2.3 for more details).

#### 2.2.1. Soil Survey through EMI Sensors

The soil variability was detected through an EMI survey on 14th June 2017, when the soil water content might be considered close to the field capacity (FC), few days after a three-day period of rainfall that resulted in 26 mm of rain (Figure 2).

The geophysical data were collected with the multi-frequency EMI sensor Profiler EMP-400 (GSSI Inc., Nashua, NH, USA). The EMI sensor worked with up to three different frequencies from 1 to 16 kHz (15 kHz, plus at most two other frequencies), corresponding to decreasing Depths of Exploration (i.e., DoE). Two frequencies were selected for the survey, 15 kHz (DoE about 1.5 m) and 10 kHz (DoE about 2.5 m), to explore the soil in contact with the vineyard’s root system, which is usually 2–3 m deep. The data were acquired along parallel rows with an interdistance of 10 m, while vineyard rows have an interdistance of 2 m. In the portions of the fields characterized by gravelly soils the EMI measurements showed not to be valid, since the EC values were found to be negative. This extreme soil texture was more present in the upper part of the soil profiles (investigated with the sensor operating at higher frequencies), and showed to have a lower weight as the depth increases.

#### 2.2.2. Vegetation and Topography Survey through UAV Multispectral and Thermal Imagery

Vegetation survey was conducted by means of an aerial campaign with sensors mounted on an UAV. The survey took place on 19th July 2017, under sunny and clear blue-sky conditions. The daily average air temperature was 26 °C, with a maximum value of 31 °C during the central hours of the day. The survey was conducted during the veraison phenological stage, in which the crop is more sensitive to crop water stress.

The UAV employed for the survey was the HexaKopter (MikroKopter, Moormerland, Germany). It is a multirotor equipped with six brushless motors, it weighs about 1.2 kg, including batteries, and its maximum transportable payload is equal to 0.5 kg. It can be remotely controlled and programmed for automatic navigation through the free and open source Mission Planner software [35]. Its maximum transmission range is about 200 m and the flight duration is limited to 10 min.

The UAV was equipped with three different sensors, in order to collect imagery in different portions of the electromagnetic spectrum: VIS (450–720 nm), NIR (800–1000 nm) and TIR (7000–14,000 nm). The Survey 2 camera (MAPIR, San Diego, CA, USA) was used for VIS acquisitions, while a modified SJ400 camera (SJCAM, Shangxue Technology Park, Putian, Shenzhen, China) was used to collect NIR imagery. Both instruments are low cost and light-weight cameras, with a CMOS sensor of maximum size 16 Mpx. TIR data were collected by the thermal camera OPTRIS PI400 (Optris GmbH, Berlin, Germany), with a spectral response in the range 7.5–13 μm. The thermal camera acquires data in radiometric video sequences format (.RAVI). For the photogrammetric processing, single frames with resolution equal to 382 × 288 px are subsequently extracted from the video. Technical specifications of the three sensors used for the vegetation survey are reported in Table 1.

**Figure 3:**
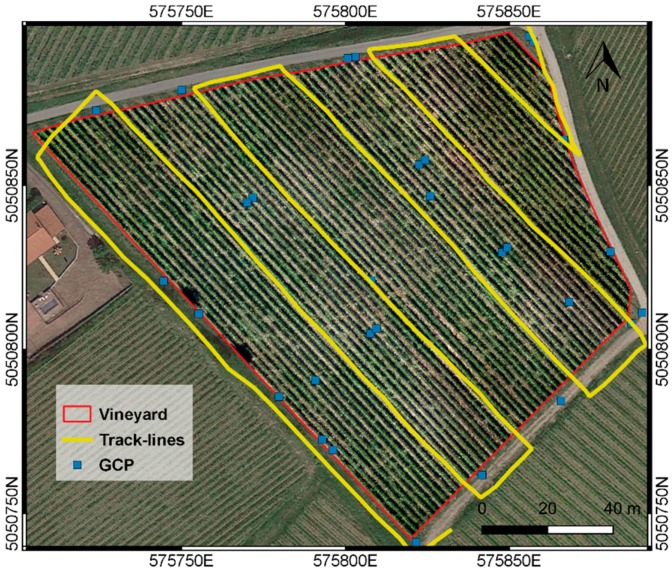
Flight track-lines for multispectral images acquisition and Ground Control Points (GCPs) distribution. Map data: ^©^Google Satellite.

According to the UAV payload, two flights were required to collect images with the three sensors. During the first flight, the UAV mounted the Survey2 and the SJ4000 cameras simultaneously, in order to acquire a multispectral dataset. Considering sensors characteristics and study area, flight planning included six strips at an altitude of 60 m above ground level (AGL), with forward and side overlaps equal to 80% and 65%, respectively. Two blocks of data (VIS and NIR) were acquired, each amounting 164 images with ground resolution, namely Ground Sample Distance (GSD), equal to 0.017 m. The adopted plan for the multispectral flight is reported in Figure 3.

During the second flight, the UAV mounted the OPTRIS PI400 to collect data of vegetation temperature. The video sequences were acquired with nadiral orientation at a constant speed of 2.7 m/s and at the altitude fixed to 55 m AGL. The derived images had a GSD of about 0.150 m and forward and side overlaps equal to 80% and 40%, respectively.

The georeferencing and the accuracy of the photogrammetric products were achieved by means of some targets used as Ground Control Points (GCPs), whose center coordinates were measured through a Global Navigation Satellite System (GNSS) receiver. A Leica Viva GS14 GNSS receiver (Leica Geosystems, Heerbrugg, Switzerland) in Network Real Time Kinematic (NRTK) mode was used in this study, with horizontal and vertical accuracies of 2–3 cm and 5 cm, respectively. Different types of targets were used for multispectral and thermal surveys: 16 black and white plastic square panels (30 cm × 30 cm) were employed for the multispectral survey, while 16 polystyrene square panels (60 cm × 60 cm), covered with aluminum foil and marked with a copper cross to enhance the central point were used for the thermal survey. In order to have an optimal distribution of GCPs, the targets were placed both all around the perimeter of the vineyard, on the ground, and inside the investigated area, on the top of the vineyard poles to ensure their visibility. Moreover, some targets with known reflectance and thermal characteristics were imaged, to perform radiometric calibration of VIS-NIR data and atmospheric correction of TIR images. According to [36], four square polystyrene panels (60 cm × 60 cm), covered with plastic, where used for the atmospheric correction of the thermal images. The panels, two white panels (i.e., cold target) and two black panels (i.e., hot target) were placed outside the investigated area in two different positions.

### 2.3. Methodological Approach for Delineating SSMZs through Data Fusion

Different types of data—geophysical data acquired through EMI sensors, and topographic and crop data acquired through the multispectral VIS-NIR sensors mounted on the UAV—were variously combined to delineate SSMZs. A data fusion approach was considered, by applying multivariate statistical methods (i.e., Principal Component Analysis, PCA) to integrate the different types of data. Precisely, the PCA was applied to the maps elaborated from geophysical and VIS-NIR data as explained in Section 3.1.

Moreover, the CWSI map was elaborated from the imagery acquired through the TIR sensor mounted on UAV. CWSI was calculated as expressed in the following formula:

(1) $$\mathrm{CWSI}=\frac{T_{s}-T_{\mathrm{wet}}}{T_{\mathrm{dry}}-T_{\mathrm{wet}}}$$

where T_S_ is the crop surface temperature, T_wet_ is the lower boundary of crop temperature corresponding to the water status of a leaf with stomata fully open and a maximum transpiration rate, T_dry_ is the upper boundary of crop temperature corresponding to the water status of a non-transpiring leaf with stomata completely closed.

The CWSI map was used to assess the effectiveness of the SSMZ delineation obtained from different combination of data even though crop yield maps are usually considered for this purpose. In this study, in absence of this type of information, the CWSI map was used as a proxy of the crop yield map. As a matter of fact, the crop water status (described through the CWSI) is assumed to be the main environmental factor affecting crop yield in this rain-fed vineyard. Areas with a low value in the CWSI map (i.e., good crop water status) were expected to correspond to areas with a high soil water content (i.e., high EC values and/or high NDVI values and/or low topographic slope values).

**Figure 4:**
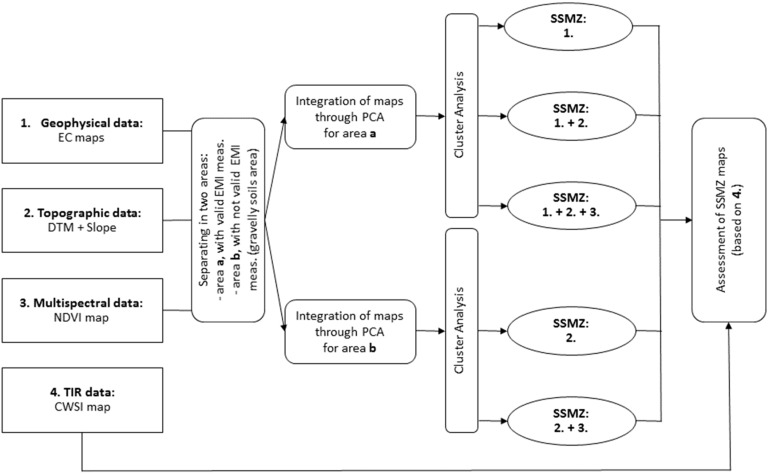
Scheme of the methodological approach adopted in this study.

Particularly, the effectiveness of the data fusion approach to enhance the delineation of SSMZs was assessed by applying the methodology hereinafter explained and illustrated in Figure 4. Two separated areas were defined within the vineyard, because of the occurrence of not valid EMI measurements for gravelly soils (Section 2.2.1). In the first area (called ‘a’), characterized with valid EMI measurements, geophysical and VIS-NIR data were available; in the second area (called ‘b’), characterized with not valid EMI measurements, only VIS-NIR data were available. For each area, maps produced from different combinations of data were fused by applying PCA. Consequently, the SSMZs were elaborated (for each area, ‘a’ and ‘b’) from the integrated maps produced through PCA (i.e., maps of the Principal Componens, PCs) by applying Cluster Analysis (CA) through the Management Zone Analyst (MZA) software [37]. MZA implements an unsupervised fuzzy classification method and determines the optimal number of SSMZs through the minimization of both the indices Normalized Classification Entropy index (NCE) and Fuzziness Performance Index (FPI); the NCE measures the degree of disorganization among zones (the larger the NCE, the higher is the amount of disorganization), the FPI measures the degree of separation between zones (the larger the FPI, the stronger is the membership sharing between zones). Specifically, the CA was applied considering only the PC maps representing most of the variability of the input maps.

For the area ‘a’, the following three cases were analyzed: (1) only geophysical data were considered: the SSMZs were delineated based on the EC maps relative to different soil depths; (2) geophysical data were considered together with the topographic data obtained from VIS-NIR imagery: elevation and slope maps as well as EC maps referred to different soil depths were used to delineate SSMZs; (3) the complete dataset including also crop data was considered: the SSMZs were delineated by integrating the NDVI map with all the previously illustrated maps. NDVI map was elaborated from VIS-NIR imagery, as the index is defined as the normalized difference between NIR and Red bands:

(2) $$\mathrm{NDVI}=\frac{\mathrm{NIR}-\mathrm{Red}}{\mathrm{NIR}+\mathrm{Red}}$$

For the area ‘b’, the following two cases were analyzed: (1) the topographic data obtained from VIS-NIR imagery were considered: elevation and slope maps were used to delineate SSMZs; (2) the complete dataset, including topographic and crop data, was considered: the SSMZs were delineated by integrating the NDVI map with elevation and slope maps.

For each case, the SSMZ map was validated through a comparison with the CWSI map. The accuracy of the correspondence between SSMZ and CWSI map was analyzed considering the distributions of CWSI values within each SSMZ.

## 3. Results and Discussion

### 3.1. Soil, Vegetation and Topography Mapping

#### 3.1.1. EC Maps

**Figure 5:**
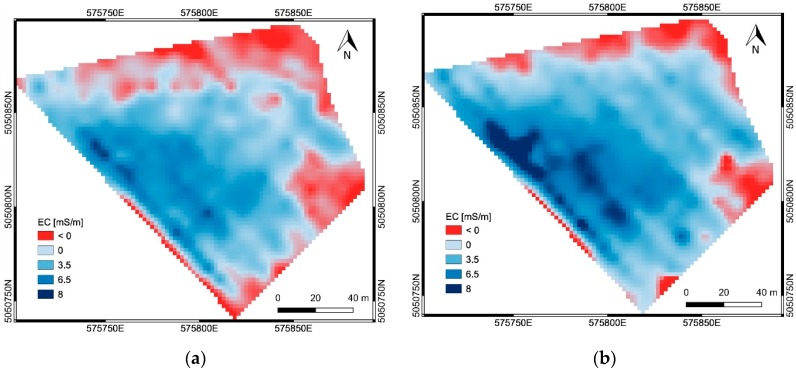
The obtained EC maps (mS/m): (**a**) frequency 15 kHz, corresponding to a DoE of 1.5 m; (**b**) frequency 10 kHz, corresponding to a DoE of 2.5 m. Red color area (area ‘b’) corresponds to gravelly soils (EMI measurement not valid).

The EC measurements obtained for each frequency used with the EMI sensor (15 kHz and 10 kHz) were interpolated on a grid with 2 m pixel size. Two EC maps were obtained (Figure 5), each one relative to a different DoE corresponding approximatively to 1.5 m and 2.5 m, respectively. 

The red color area (area ‘b’) in each map of Figure 5 corresponds to gravelly soils, for which the EMI measurements were not valid, because of the very low EC values characterizing those soils. In these zones, negative EC values were obtained from the EMI survey. The total extent of these areas decreases with the increasing DoE.

#### 3.1.2. Topography and Slope Maps

The VIS and NIR imagery blocks were processed through standard photogrammetric workflow [38] with the Agisoft Photoscan Professional software v. 1.2.6 [39]. Finally, the Digital Surface Model (DSM) was produced with a spatial resolution equal to 0.05 m, representing the height model for both vegetation and soil.

**Figure 6:**
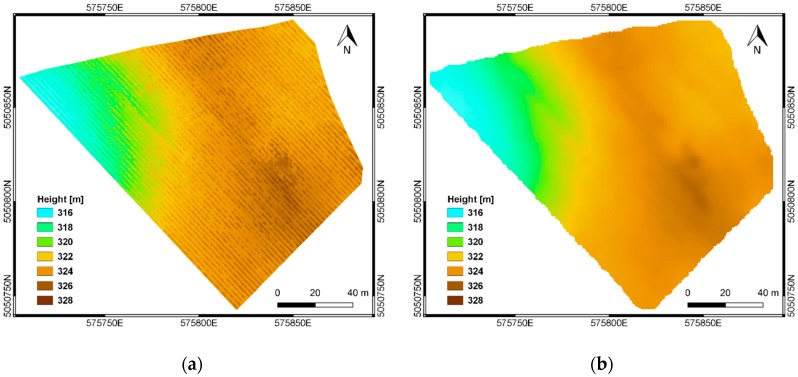
DSM (**a**) and DTM (**b**) produced through photogrammetric processing of multispectral (VIS-NIR) dataset.

In order to reconstruct the soil topography, vegetation pixels were detected and removed from the DSM. Vegetation detection was performed on the DSM by using an algorithm developed by the authors, which assumes that pixels with higher elevation values correspond to vegetation [40]. The algorithm classifies as vegetation pixels all the pixels having a height value greater than a user-defined threshold within a moving window. In a second step, vegetation pixels were subtracted from the DSM, thus producing a model representing the height of the terrain, namely the Digital Terrain Model (DTM) of the study area. Figure 6 shows the DSM and the DTM of the vineyard, obtained after photogrammetric processing and vegetation detection and removal, respectively. The final DTM reported in Figure 6b, obtained after the application of a moving average smoothing filter, has a spatial resolution of 2 m.

**Figure 7:**
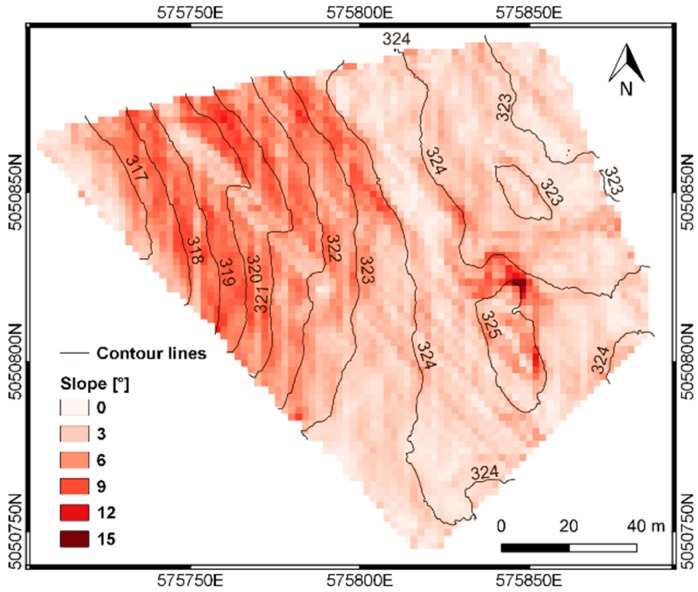
Slope map and contour lines derived from the DTM.

Slope and contour line maps were derived from the DTM, by using the *Raster Terrain Analysis* functions in QGIS v 3.2 [41]. A fixed interval of 1 m was set for the generation of the contour lines, while the slope map was computed as the gradient of the terrain model, having the same spatial resolution of the DTM (i.e., 2 m). Final results are shown in Figure 7.

#### 3.1.3. Vegetation Indices Maps (NDVI and CWSI)

**Figure 8:**
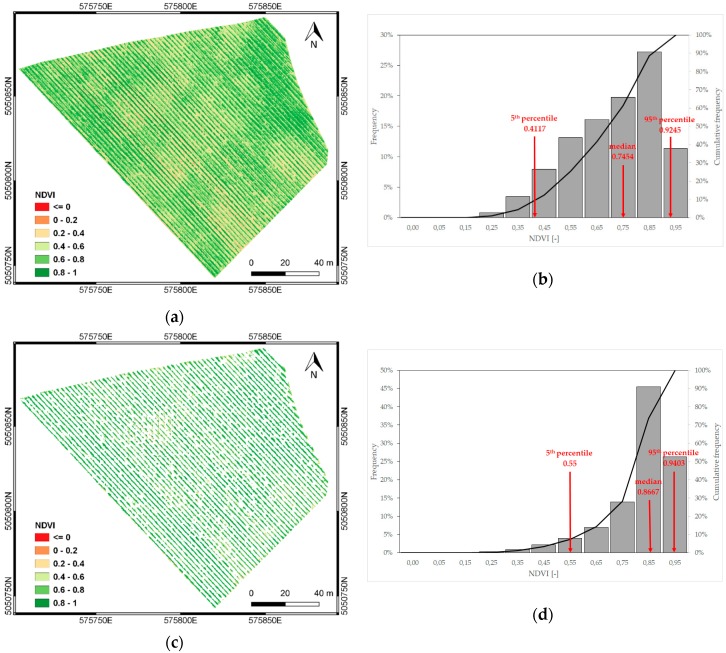
NDVI map before (**a**) and after (**c**) soil masking, together with their respective frequency distribution (**b**,**d**).

Multispectral VIS-NIR and TIR imagery were used to compute the vegetation indices NDVI and CWSI, commonly adopted to describe vegetation vigor and crop water status, respectively. A VIS-NIR orthomosaic was generated in Digital Number (DN) with a spatial resolution of 0.05 m, then converted in reflectance values, through the radiometric calibration obtained with an empirical line correction approach [42]. Images of the radiometric targets were used to compute the linear regression coefficients of the DN values against the reflectance values from the target surface. The orthomosaic corrected through the radiometric calibration was used to obtain the NDVI map (Equation (2)). Moreover, soil was masked out, to avoid the inclusion of soil pixels in the vegetation maps. The soil mask was created by extracting pixels that were not considered to be vegetation, as described in Section 3.1.2. Figure 8 shows NDVI maps before and after the soil pixel removal. From the graphs showing the frequency distribution of the NDVI maps (Figure 8b,d), it is evident that most of the pixels with NDVI values lower than 0.7-corresponding to soil and inter-row grass—could be removed and only vegetation pixels (NDVI values greater than 0.7) were retained.

The CWSI map was obtained from the TIR orthomosaic. The TIR orthomosaic (with spatial resolution equal to 0.15 m) was generated through a specific procedure for TIR images. This procedure, including single frames extraction, format conversion and photogrammetric processing, is described in detail in [43]. Atmospheric correction of the obtained TIR orthomosaic was performed by using the thermal images of the cold and hot targets, representing respectively the minimum (*T_min_*) and the maximum (*T_max_*) temperature values within the investigated area. The temperature of the targets was recorded at the ground level as well. These temperature values, acquired at flight height and at ground level, were used to derive an atmospheric model [36] successively applied to the TIR orthomosaic to obtain the crop surface temperature (called crop surface TIR orthomosaic hereinafter).

**Figure 9:**
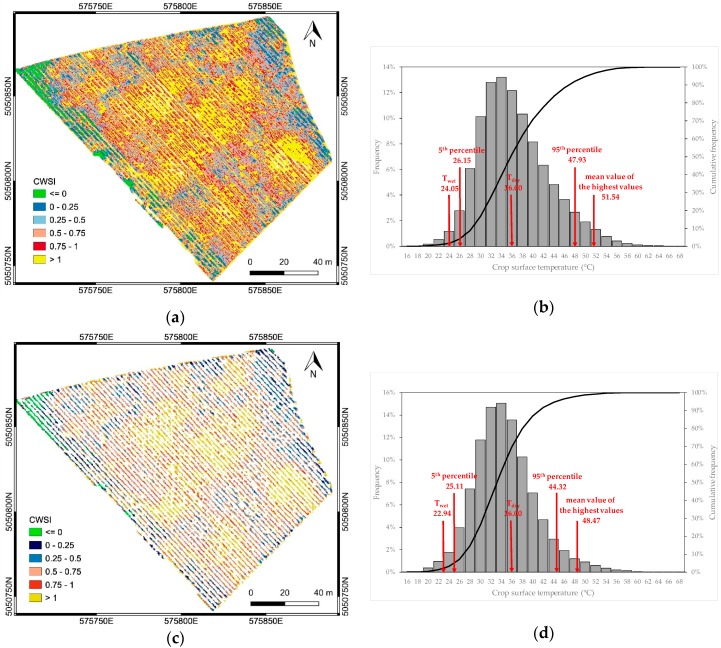
CWSI map before (**a**) and after (**c**) soil masking. The frequency distribution of the crop surface temperatures, with the illustration of T_wet_ and T_dry_ values calculated according to the empirical approach described in Section 3.1.3, is reported for each case (**b**,**d**).

The CWSI map was calculated using Equation (1). The values T_wet_ and T_dry_ were initially determined by an empirical approach reported in many studies [44,45,46,47]. The value T_dry_ was calculated by using the current T_air_ plus 5 °K [48,49,50], while the value T_wet_ was calculated as the mean of the coolest 5% vegetated pixels in the crop surface TIR orthomosaic. As for the NDVI map, soil was masked out from the crop surface temperature map. Figure 9 illustrates the CWSI maps before and after soil pixels removal. 

**Figure 10:**
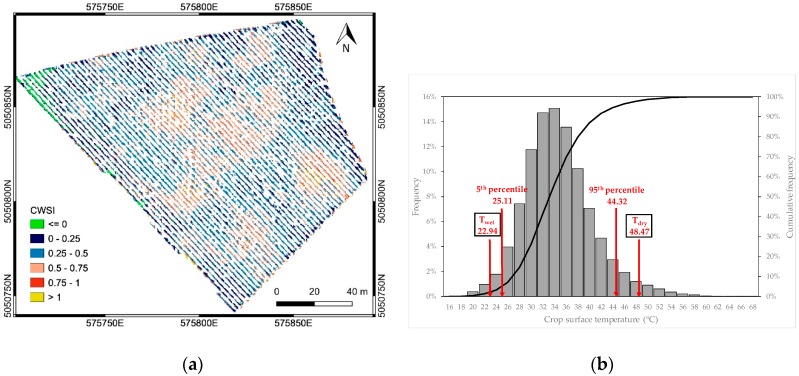
CWSI map after soil masking (**a**), derived considering the T_wet_ and T_dry_ values calculated as the mean of the coolest 5% and the hottest 5% vegetated pixels in the crop surface TIR orthomosaic, respectively. The frequency distribution of the crop surface temperatures is also reported (**b**).

Both maps show a zone with values greater than 1, due to the presence of pixels with surface temperature higher than T_dry_. This could be due to the fact that, in this study, T_air_ (31°C) was the average hourly temperature registered during the central hours of the day (from 1:00 p.m. to 2:00 p.m., solar time) at the Erbusco agro-meteorological station, placed 2 km away from the experimental site and positioned over a standard grass surface as indicated by WMO (World Meteorological Organization). In order to estimate a more reliable value for T_dry_, the same approach used for T_wet_ was adopted: T_dry_ was calculated based on the temperature histogram [51,52,53] as the mean of the hottest 5% vegetated pixels in the crop surface TIR orthomosaic. The derived CWSI map, successively used in this study, is shown in Figure 10.

### 3.2. SSMZ Mapping

Firstly, the SSMZ map was elaborated from EC maps only (Section 3.2.1). Afterwards, topography and crop information were integrated with the EC maps through PCA, to improve the SSMZ map (Section 3.2.2 and Section 3.2.3). The Pearson’s correlation coefficients were computed separately for areas ‘a’ and ‘b’, respectively among the EC, DTM, Slope and NDVI values calculated at the grid nodes used to interpolate the EC data (2 m pixel size), with valid EMI measurements (Table 2), and among the DTM, Slope and NDVI values calculated at the nodes of the same grid, with not valid EMI measurements (Table 3). All the coefficient values are statistically significant with level 0.001 (*p*-value < 5 × 10^−4^). Moreover, the Moran Index was calculated to describe the spatial autocorrelation of the variables and the spatial cross-correlation between the variables. The values, calculated separately for areas ‘a’ and ‘b’, considering the nodes respectively inside and outside the vineyard’s area with valid EMI measurements, are reported respectively in Table 4 and Table 5. The Moran Index values were always statistically significant with level 0.001 (*p*-value < 5 × 10^−4^). The univariate Moran Index calculated for the different variables was always positive and greater than 0.60, showing a high spatial autocorrelation of all the variables. The bivariate Moran Index (between variables) describes the correlation based on the relationships between each point and the neighboring ones. According to [54], the correlation described through the Pearson’s coefficients can be decomposed in two components, related to a direct correlation without distance effect and an indirect correlation based on the distance effect. Consequently, the difference between the Pearson’s coefficient and the Moran Index quantifies the direct correlation component. For the two datasets described in Table 4 and Table 5, this difference was always less than 0.03 in absolute value, equal to the 25% of the correlation coefficient at most, except for the correlation between the variables EC-15 kHz and EC-10 kHz (Table 4) and between the variables DTM and Slope (Table 5). These results highlighted the relevant contribution of the spatial pattern in cross-correlation.

#### 3.2.1. Delineation of SSMZs from EC Maps

**Figure 11:**
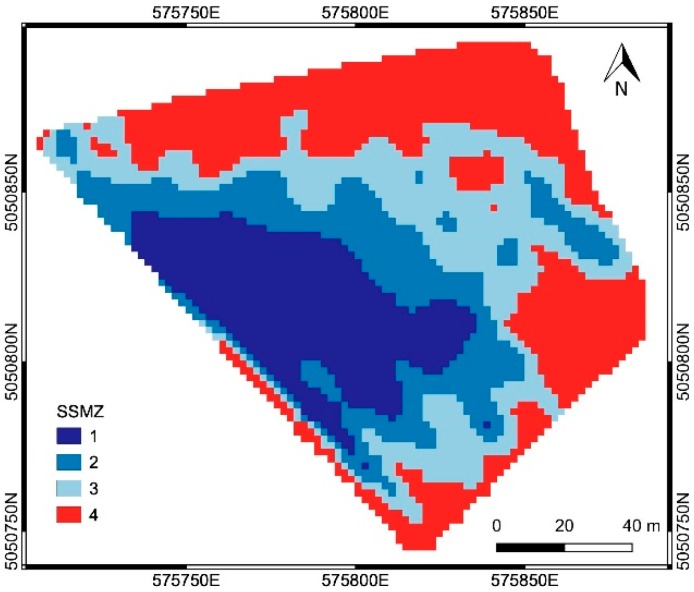
The SSMZ map obtained from the EC maps relative to frequencies 15 kHz and 10 kHz. SSMZ from 1 to 4 corresponds to decreasing EC values; in particular, SSMZ 4 corresponds to negative EC values, due to gravelly soils.

The two EC maps relative to frequencies 15 kHz and 10 kHz, calculated within area ‘a’ considering only the valid EMI measurements (i.e., not negative EC values), were analyzed through the PCA. The SSMZ map (Figure 11) was obtained by applying CA to the first PC, explaining about 96% of the variability of both the EC maps. Three SSMZs were delineated within area ‘a’; another SSMZ (red color) was defined, corresponding to area ‘b’ characterized with not valid EMI measurements (i.e., negative EC values) occurred for gravelly soils (Figure 5). SSMZs from 1 to 4 (Figure 11) correspond to decreasing EC values.

SSMZ and CWSI maps were compared. High EC values (i.e., high soil water contents) were expected to correspond with low CWSI values (i.e., good crop water status). Instead, areas with high CWSI values, denoting crop water stress, were included in the SSMZ 1 (characterized by high EC values), while, vice-versa, areas with low CWSI values were present in the SSMZ 4 (very low EC values). The analysis showed that for the study vineyard, physical-chemical soil properties described by the EC values where not sufficient to explain the crop water status. As matter of fact, the spatial patter of the SSMZs is quite different from that one of the zones in the CWSI map correspondent to low index values (from 0 to 0.5) and high index values (from 0.5 to 1).

#### 3.2.2. Data Fusion: Delineation of SSMZs from Slope and EC Maps

The combined effect of soil properties (i.e., EC values) and field topography (i.e., elevation and slope) was investigated to improve the delineation of SSMZs, looking for a better correspondence with the spatial distribution of the zones in CWSI map with low and high index values. EC, elevation and slope maps were analyzed through PCA. The SSMZ map was obtained by applying CA to the first and second PCs explaining most of the variability of all the considered maps. Particularly, PCA and CA were applied separately to the area ‘a’ with valid EMI measurements, corresponding to SSMZs from 1 to 3 in Figure 11, as well as to the gravelly soil area ‘b’, corresponding to SSMZ 4 in Figure 11.

**Figure 12:**
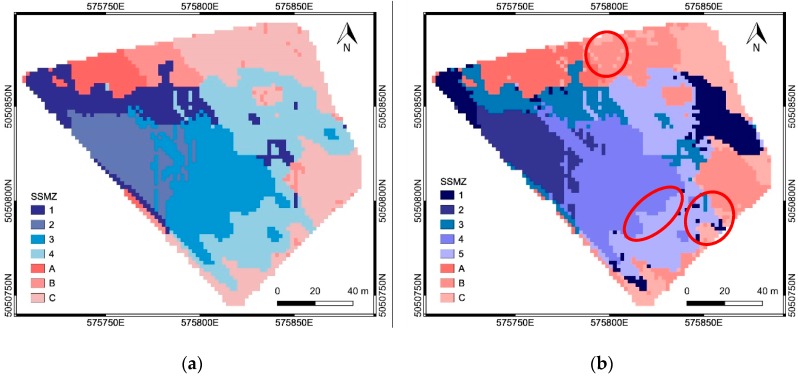
The SSMZ map obtained from: (**a**) EC, elevation and slope maps; (**b**) EC, elevation, slope and NDVI.

**Figure 13:**
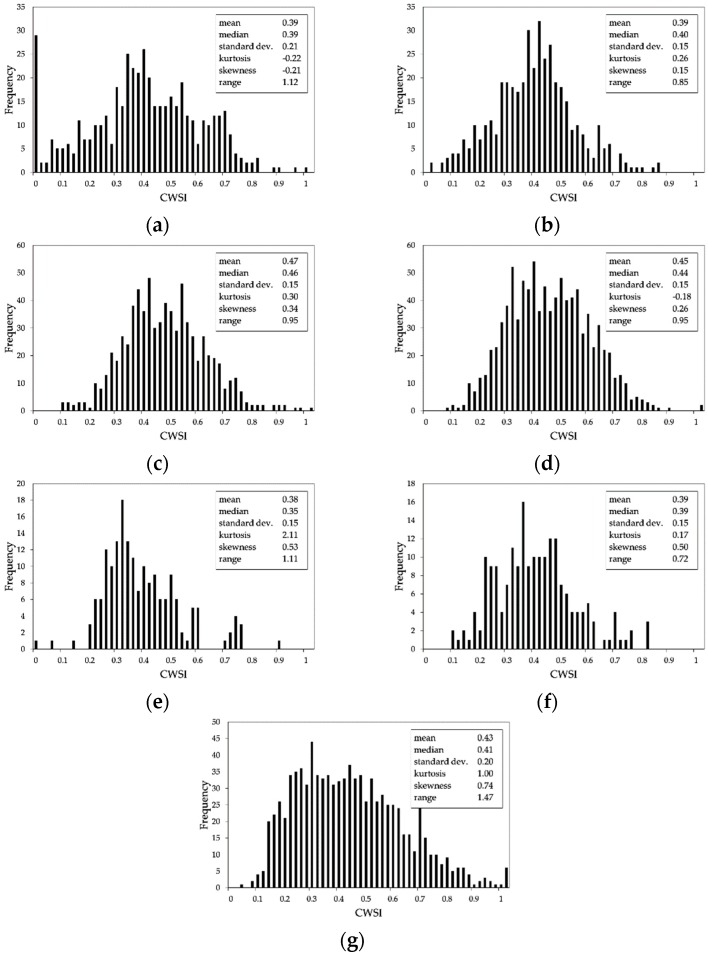
Distribution of the CWSI values within each SSMZ shown in Figure 12a: (**a**) SSMZ 1; (**b**) SSMZ 2; (**c**) SSMZ 3; (**d**) SSMZ 4; (**e**) SSMZ A; (**f**) SSMZ B; (**g**) SSMZ C.

In the former area, four SSMZs (numbered from 1 to 4 in Figure 12a), were delineated considering EC, elevation and slope maps. In the latter area, three SSMZs (numbered from A to C in Figure 12a) were recognized taking into account only elevation and slope maps. The resulting SSMZ map is shown in Figure 12a. This map, even though improved with respect to that obtained from EC maps only (Figure 11), could not completely explain the spatial variability detected in the CWSI map. Indeed, as illustrated also in Figure 13 showing the distributions of the CWSI values within each SSMZ, the SSMZs 1 and 2 (high EC values) corresponded to low CWSI values (as expected), as well as for the SSMZ A and part of the SSMZ B; on the other hands, SSMZs 3 and 4 included areas with both low and high CWSI values (not expected due to the low EC values), as for the cases of SSMZ C and part of B. This behavior highlighted how the zonation shown in Figure 12a did not consider all the factors affecting the crop water status.

#### 3.2.3. Data Fusion: Delineation of SSMZs from Soil Maps (Slope and EC Maps) and NDVI Map

Finally, also the variability of crop vigor (described by the NDVI map) was taken into account to produce a more reliable SSMZ map, with better correspondence to the zones in the CWSI map characterized by high (from 0.5 to 1) and low values (from 0 to 0.5) of the index. EC, elevation, slope and NDVI maps were analyzed through PCA and CA. Following the same approach considered in the Section 3.2.2, the SSMZ map was obtained by applying PCA and CA firstly to data available within the area ‘a’ with valid EMI measurements, and afterwards to data available within the area ‘b’ characterized by gravelly soils.

For area ‘a’, CA was applied to the first three principal components PC^a^_1_, PC^a^_2_ and PC^a^_3_ (Table 6), obtained from the EC, elevation, slope and NDVI maps: PC^a^_1_ represented mainly the physical-chemical soil properties (correlation coefficients with EC greater than 0.90), and partly the DTM (correlation coefficient equal to −0.48); PC^a^_2_ represented the topography (correlation coefficients with DTM and Slope equal to 0.72 and −0.45, respectively); PC^a^_3_ represented both the topography (correlation coefficient with Slope equal to −0.61) and the crop vigor (correlation coefficient with NDVI equal to 0.59) which are negatively correlated (see Table 2). For area ‘b’, CA was applied to the first two principal components PC^b^_1_ and PC^b^_2_ (Table 7), obtained from the elevation, slope and NDVI maps: PC^b^_1_ represented the topography (correlation coefficients with DTM and Slope equal to −0.89 and 0.88, respectively); PC^b^_2_ represented the crop vigor (correlation coefficient with NDVI equal to 0.99).

**Figure 14:**
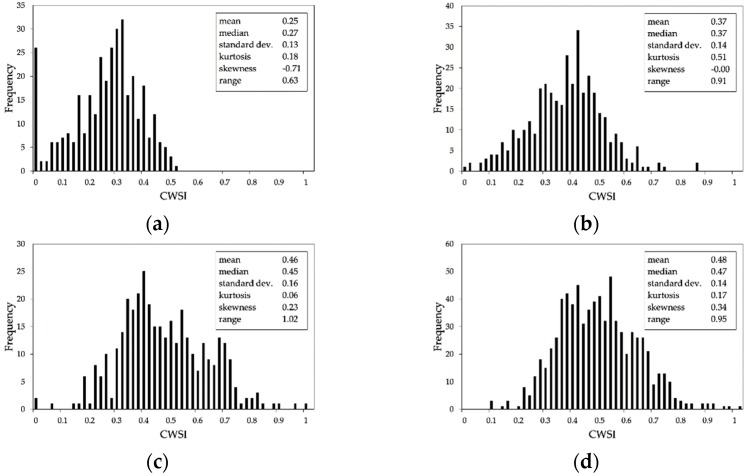

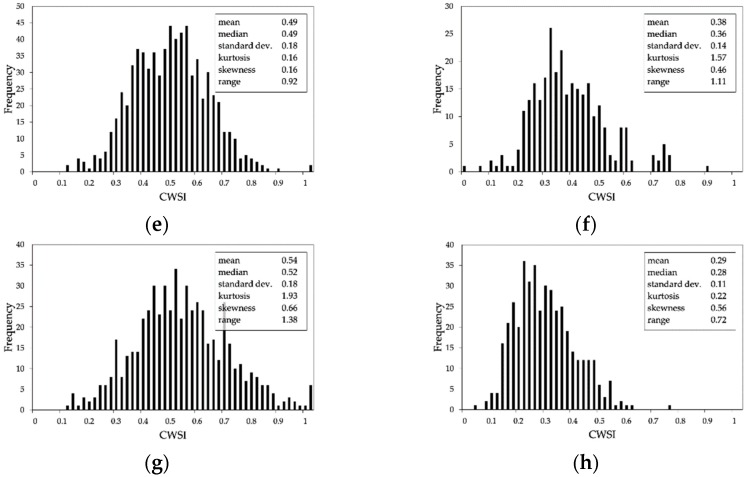
Distribution of the CWSI values within each SSMZ shown in Figure 12a: (**a**) SSMZ 1; (**b**) SSMZ 2; (**c**) SSMZ 3; (**d**) SSMZ 4; (**e**) SSMZ 5; (**f**) SSMZ A; (**g**) SSMZ B; (**h**) SSMZ C.

Within areas ‘a’ and ‘b’, respectively, five SSMZs (numbered from 1 to 5) were delineated from EC, elevation, slope and NDVI maps, and three SSMZs (numbered from A to C) were delineated considering only elevation, slope and NDVI maps. The resulting SSMZ map is shown in Figure 12b. The SSMZs 1–3, A and C corresponded to low CWSI values, while SSMZs 4, 5, and B mostly corresponded to high CWSI values, except for the three small areas highlighted with the red circles in Figure 12b. As matter of fact, the SSMZ delineation in Figure 12b was improved compared to the one shown in Figure 12a, as illustrated in Figure 14: (i) the mean and the standard deviation of the CWSI values within SSMZ 1 decreased, while SSMZs 5 (corresponding to SSMZ 4 in Figure 12a) mostly included high CWSI values, with an increased mean value compared to SSMZ 4 in Figure 12a; (ii) also the mean of the CWSI values within SSMZs 3 increased with respect to the values for SSMZ 1 in Figure 12a; (iii) the SSMZ B was characterized by the CWSI values with the highest mean; (iv) the SSMZ C mostly included low CWSI values (the mean and the standard deviation of the CWSI values within this SSMZ decreased compared to the values for SSMZ C in Figure 12a). Finally, the integration of the NDVI data allowed the delineation of SSMZs each one corresponding respectively to low or high CWSI values (except for the three small areas highlighted with red circles in Figure 12b).

**Figure 15:**
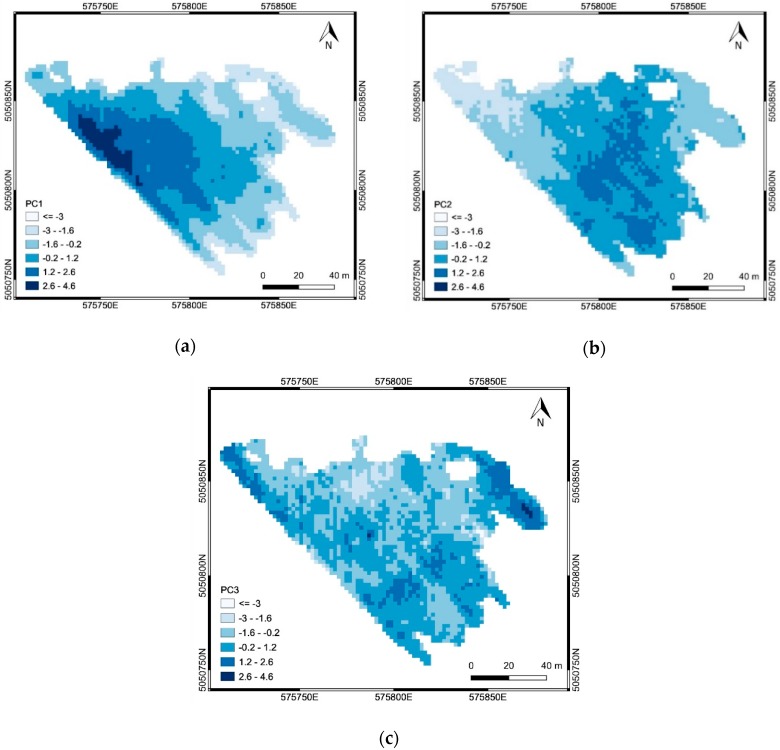
Results of the PCA applied in the area ‘a’: spatial distribution of PC^a^_1_ (**a**), PC^a^_2_ (**b**) and PC^a^_3_ (**c**).

**Figure 16:**
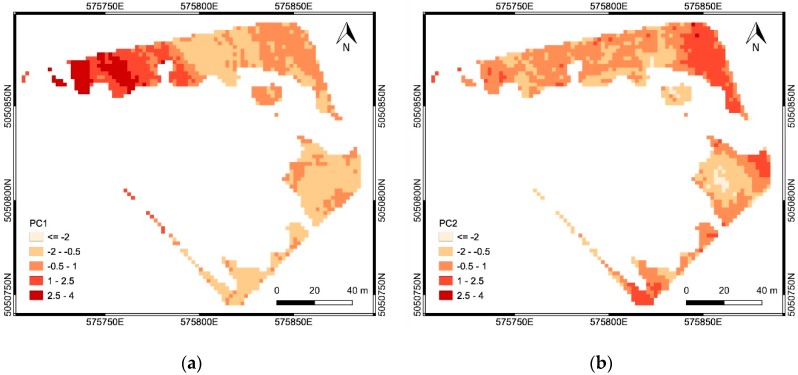
Results of the PCA applied in the area ‘b’: spatial distribution of PC^b^_1_ (**a**), PC^b^_2_ (**b**).

The spatial distributions of the PCs (Figure 15 and Figure 16) explain which factors prevailed in the SSMZ delineation through CA. The SSMZs 1-3 were mainly determined by soil properties (EC data, described by PC^a^_1_), while the SSMZ 4, as well as the SSMZs A and B, were mainly determined by topography (DTM and Slope data, described by PC^a^_2_ for the case of SSMZ 4, and by PC^b^_1_ for the case of SSMZs A and B). The SSMZs 5 and C were mainly determined by crop vigor (NDVI data, described by PC^a^_3_ for the case of SSMZ 5, and by PC^b^_2_ for the case of SSMZ C).

Moreover, Table 8 shows the correlation between CWSI and the variables (elevation, slope and NDVI) used to integrate the EMI measurements in order to improve the reliability of the SSMZ map obtained from only EC data. Correlation with NDVI was the highest (*p*-values less than 5 × 10^−4^), highlighting how NDVI data were able to explain the CWSI spatial variability within the whole field area. As matter of fact, for NDVI the difference between the Pearson’s coefficient and the Moran Index, quantifying the direct correlation component independent from the spatial variability, is almost −0.30, while this component is almost zero for the other variables.

### 3.3. Discussion

Obtaining a reliable SSMZ map is of practical relevance for farmers, since this map is an important tool to actuate variable rate practices in PA, in term of both designing and managing application systems (e.g., for the water and nutrient management). As matter of fact, the delineated SSMZs are zones where the factors influencing the crop yield (i.e., soil, topography, and micro-climate) result in affecting the crop water status and vigor in a different way. These factors need to be adequately described through thematic maps, to allow the delineation of SSMZs through their combination.

This work proposed a fusion approach integrating different thematic maps (EC, DTM, slope and NDVI maps) to compute a SSMZ map, whose effectiveness was assessed considering a CWSI map. The approach was applied in a rainfed vineyard to obtain a SSMZ map useful for the design and the management of a variable rate irrigation system. By actuating a variable rate irrigation accordingly to the SSMZ map, farmers would achieve a twofold result: first, to obtain a higher and more uniform production and second, to optimize the water use. Interesting general discussion points that emerge from the results of this study are the following:

(1) the SSMZ map can vary greatly its spatial configuration depending on the information layers used for its production, it is therefore necessary to conduct more research aimed at understanding which information it may be appropriate to include, based not only on the prevailing factors affecting the crop yield, but also on the purpose for which the SSMZ map is being developed (e.g., nutrient management, water management);
(2) the addition of the topographic information to the soil data included in the EC maps leads the SSMZ map to have a spatial distribution more similar to that shown by the CWSI map; it can be deduced that in a vineyard in slope conditions, the topographic information together with the soil distribution information are able to explain part of the variability illustrated in the vegetation maps;
(3) in the specific case of this study (i.e., rain-fed vineyard in severe water stress conditions), NDVI data were able to explain the CWSI spatial variability within the whole field area; indeed, the NDVI map showed a strong correlation with the crop water status (CWSI);
(4) while information on soil properties and topography are not very variable over time, crop data may vary from year to year if soils and topography are not the only factors conditioning their distribution; it would therefore be necessary to repeat the study for several years to verify the ‘stability over time’ of the spatial distribution of crop data;
(5) if the SSMZ map is to be used to design a ‘rigid’ variable-rate irrigation system (i.e., drip irrigation system subdivided into different irrigation sectors), considering that the geometry of the irrigation system (and consequently the irrigation amounts distributed) cannot be changed from year to year, it is even more important to verify the ‘stability over time’ of the spatial distribution of crop data, and if therefore it makes sense to consider them in the delineation of the SSMZs.

## 4. Conclusions

Recent literature suggests that integrating EC information with elevation and slope maps, as well as with crop indices describing the crop vigor, can improve the delineation of SSMZs in vineyards. This was demonstrated in this study, focusing on the fusion of EC maps obtained by an EMI geophysical survey and VIS-NIR data collected through UAV-mounted cameras, to optimally delineate SSMZs in a rain-fed vineyard of 15,000 m^2^ located in Northern Italy. In the study, in absence of a spatially distributed crop yield map, the crop water status detected in a severe dry period during the grape’s veraison phenological stage was assumed to summarize the effect of the principal environmental factors acting on the crop production; consequently, the CWSI map was used as a proxy of the crop yield map itself.

The obtained results stressed how the crop water status in the study vineyard was actually affected significantly not only by the physical-chemical soil properties (described by the EC maps), but also by the elevation and slope of terrain. Moreover, the NDVI map allowed us to include in the analysis time-dependent factors influencing the production (i.e., interaction among soil, topography, micro-climate and vegetation). In fact, for the study vineyard, a good correspondence between the spatial pattern of SSMZ and CWSI maps was achieved only by integrating the NDVI data to the other types of data. Consequently, at least for the study case, a reliable SSMZ map to be used to design and manage irrigation within the vineyard showed to require the availability of both ‘stable-over-time information’, related to soil properties and topography, and ‘time-dependent information’, related to the crop development. In this study, crop information was available only for the 2017 season, but a good practice to obtain reliable SSMZ maps would require the acquisition of crop data during different seasons.

## Figures and Tables

**Table 1:** Technical specifications of the three cameras used for the vegetation survey.

	Survey 2	SJ4000	OPTRIS PI400
Acquisition	VIS	NIR	TIR
Focal length (mm)	4.35	4.35	8
Sensor size (mm)	4.86 × 3.64	4.86 × 3.64	9.55 × 7.2
Sensor size (px)	4032 × 3024	4032 × 3024	382 × 288
Pixel size (μm)	1.2	1.2	25
Field of View (FOV)	82°	82°	62° × 49°
Output format	JPEG image	JPEG image	RAVI video
Weight (g)	64	64	380

**Table 2:** Pearson’s correlation coefficients among the variables used to delineate SSMZ, estimated considering the grid nodes with valid EMI measurements (area ‘a’).

	EC-15 kHz	EC-10 kHz	DTM	Slope	NDVI
**EC-15 kHz**	1	0.93 ***	−0.20 ***	0.23 ***	−0.22 ***
**EC-10 kHz**		1	−0.29 ***	0.26 ***	−0.21 ***
**DTM**			1	−0.40 ***	−0.19 ***
**Slope**				1	−0.07 **
**NDVI**					1

*** *p*-value < 5 × 10^−5^; ** *p*-value < 5 × 10^−4^.

**Table 3:** Pearson’s correlation coefficients among the variables used to delineate SSMZ, estimated considering the grid nodes with not valid EMI measurements (area ‘b’).

	EC-15 kHz	EC-10 kHz	DTM	Slope	NDVI
**EC-15 kHz**	-	-	-	-	-
**EC-10 kHz**		-	-	-	-
**DTM**			1	−0.40 ***	−0.12 **
**Slope**				1	−0.08 *
**NDVI**					1

*** *p*-value < 5 × 10^−5^; ** *p*-value < 5 × 10^−4^; * *p*-value < 5 × 10^−3^.

**Table 4:** Moran Index among the variables used to delineate SSMZ, estimated (using GeoDa software, by Luc Anselin) considering the grid nodes with valid EMI measurements (area ‘a’).

	EC-15 kHz	EC-10 kHz	DTM	Slope	NDVI
**EC-15 kHz**	0.82 **	0.79 **	−0.20 **	0.23 **	−0.24 **
**EC-10 kHz**		0.81 **	−0.28 **	0.27 **	−0.22 **
**DTM**			0.99 **	−0.40 **	−0.19 **
**Slope**				0.63 **	−0.07 **
**NDVI**					0.76 **

*** *p*-value < 5 × 10^−5^; ** *p*-value < 5 × 10^−4^.

**Table 5:** Moran Index among the variables used to delineate SSMZ, estimated (using GeoDa software, by Luc Anselin) considering the grid nodes with not valid EMI measurements (area ‘b’).

	EC-15 kHz	EC-10 kHz	DTM	Slope	NDVI
**EC-15 kHz**	-	-	-	-	-
**EC-10 kHz**		-	-	-	-
**DTM**			0.96 **	−0.59 **	−0.15 **
**Slope**				0.69 **	−0.09 **
**NDVI**					0.66 **

*** *p*-value < 5 × 10^−5^; ** *p*-value < 5 × 10^−4^; * *p*-value < 5 × 10^−3^.

**Table 6:** Results of PCA applied in the area ‘a’: variance of the principal components considered in CA and correlation coefficients with the variables used to delineate SSMZ.

	Variance	Cumulative Variance	EC-15 kHz	EC-10 kHz	DTM	Slope	NDVI
**PC^a^_1_**	2.26	45%	0.90	0.93	−0.48	0.52	−0.27
**PC^a^_2_**	1.29	71%	0.25	0.18	0.72	−0.45	−0.69
**PC^a^_3_**	0.87	88%	0.28	0.26	0.03	−0.61	0.59

**Table 7:** Results of PCA applied in the area ‘b’: variance of the principal components considered in CA and correlation coefficients with the variables used to delineate SSMZ.

	Variance	Cumulative Variance	DTM	Slope	NDVI
**PC^b^_1_**	1.56	52%	−0.89	0.88	0.06
**PC^b^_2_**	1.03	87%	−0.14	−0.20	0.99

**Table 8:** Correlation between CWSI and variables DTM, Slope and NDVI.

	DTM	Slope	NDVI
**Pearson’s coefficient**	0.29 ***	0.02 *	−0.71 ***
**Moran Index**	0.29 **	0.02 **	−0.52 **

*** *p*-value < 5 × 10^−5^; ** *p*-value < 5 × 10^−4^; * *p*-value > 0.1.
